# Insufficiency Fractures of the Iliac Crest Following Robot-Assisted Total Hip Arthroplasty: A Report of Two Cases

**DOI:** 10.7759/cureus.87358

**Published:** 2025-07-05

**Authors:** Tetsuya Tachibana, Hiroki Katagiri, Toshifumi Watanabe, Ryusuke Saito, Tetsuya Jinno

**Affiliations:** 1 Department of Orthopaedic Surgery, Dokkyo Medical University Saitama Medical Center, Saitama, JPN; 2 Department of Joint Surgery and Sports Medicine, Institute of Science Tokyo, Tokyo, JPN

**Keywords:** iliac crest, insufficiency fractures, mako, robot-assisted surgery, stress-fracture, total hip arthroplasty

## Abstract

The risk of insufficiency fractures at the iliac crest following pin insertion during robot-assisted total hip arthroplasty (THA) is unknown, as there have been very few reports on this complication. Here, we report two cases of insufficiency fractures of the contralateral iliac crest following robot-assisted THA using the Mako system (Stryker Orthopaedics, Mahwah, NJ, USA). Both patients underwent left THA using the anterolateral supine approach, and three threaded bone pins (4.0 mm diameter) were inserted into the right iliac crest for pelvic array fixation. In case one, all three pins achieved bicortical fixation. In case two, one pin demonstrated long transcortical fixation with the outer cortex of the ilium, another was inserted into soft tissue, and the third pin was fixed monocortically. Postoperatively, both patients were discharged without pain or radiographic evidence of fracture; however, contralateral iliac pain developed approximately four weeks postoperatively without trauma. Insufficiency fractures of the iliac crest at the pin insertion sites were confirmed by plain radiography. Bone union was observed within three to six months of conservative treatment in both cases, with T-cane ambulation and no weight-bearing restrictions. These cases suggest that both bicortical and transcortical pin fixation to the iliac crest may cause insufficiency fractures of the iliac bone. This report highlights the need for increasing awareness of insufficiency fractures associated with pin insertion in robot-assisted THA.

## Introduction

Accurate acetabular component placement is crucial for preventing aseptic loosening and dislocation in total hip arthroplasty (THA) [1]. However, precise cup placement requires a learning curve and is a technically demanding procedure [2]. The safe zone proposed by Lewinnek et al. of -40° ± 10° of inclination and 15° ± 10° of anteversion is widely cited as a guideline for optimal cup alignment [3]. However, recent simulation studies have shown that to avoid implant impingement, the actual safe zone must be more narrowly defined based on parameters such as femoral stem anteversion [4,5]. Therefore, precise and individualized cup placement is essential. Semi-active robot-assisted THA via the Mako system (Stryker Orthopaedics, Mahwah, NJ, USA), a recent introduction, enables precise acetabular reaming and cup placement with robotic assistance [6].

While the use of the Mako has been reported to improve cup placement accuracy, a few reports on intraoperative and postoperative complications have been documented, with only a few studies mentioning prolonged operative times due to pin placement and registration [6]. To achieve accurate registration with the Mako system based on CT-based modeling, three bone pins (4 mm diameter) are typically inserted into the iliac crest to fix the pelvic array. Surface registration is performed according to the Mako system's instructions, targeting areas such as the external iliac plate, the anterior periarticular region, and the intra-acetabular region. During acetabular reaming and cup placement, the robot haptic arm guides the reamer, while the cup impactor ensures alignment with the preplanned placement targets. Various complications may occur in the early stages of the adoption of new technology. This report describes two cases in which patients developed pain at the contralateral iliac crest several weeks after Mako-assisted THA and were diagnosed with insufficiency fractures at the iliac crest.

## Case presentation

Case one

**Figure 1:**
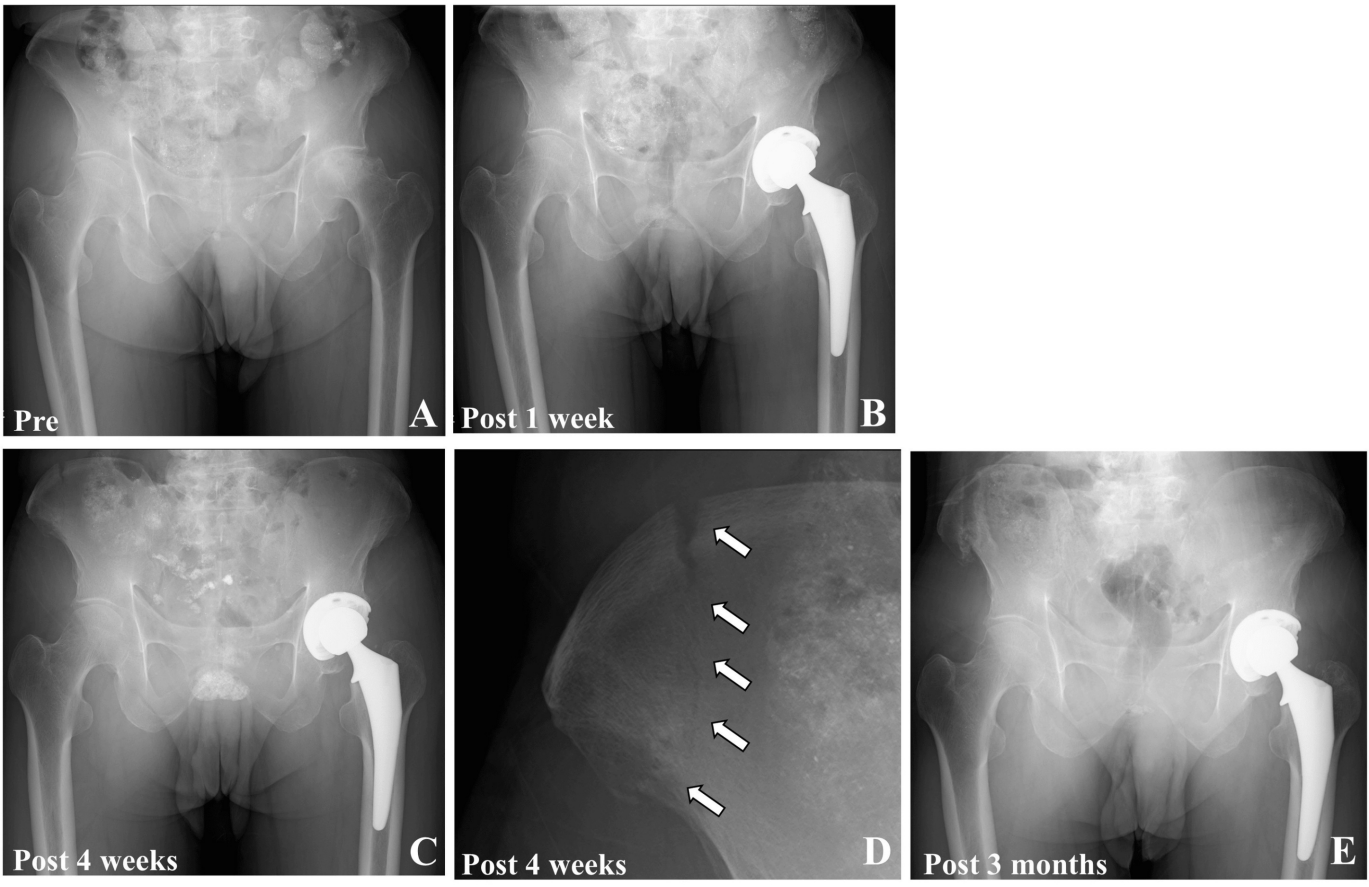
Pre and postoperative X-rays of case one A: Preoperative pelvic X-ray diagnosed as left femoral head osteonecrosis; B: Postoperative X-ray one week after surgery; C: X-ray at four weeks postoperatively showing stress fracture of the right iliac crest; D: Enlarged view of the right ilium from panel C. The white arrow indicates the fracture line of the iliac crest. E: Postoperative X-ray after three months showing slight displacement and bone union of the iliac crest fracture.

The patient was a 60-year-old male (height, 169 cm; weight, 63 kg) with a history of hypertensive nephropathy on maintenance dialysis. He had no history of alcohol consumption or steroid use and had not received any treatment for fragility fractures or osteoporosis. We diagnosed left idiopathic osteonecrosis of the femoral head based on radiography (Figure 1A) and MRI, and classified the osteoarthritic change as grade 3 according to the Kellgren-Lawrence classification. Left THA was performed. The preoperative bone mineral density (BMD), assessed using the T-score, was 0.7 in the lumbar spine (L1-L4) and −2.8 in the proximal left femur. The procedure was performed in the supine position under general anesthesia using the anterolateral supine (ALS) approach [7]. Three 5 mm skin incisions were made in the right (contralateral) iliac crest. A 4 mm bone pin was first inserted using a power drill and freehand technique. A three-pin pelvic clamp was then used to guide the alignment and ensure proper spacing of the second bone pin, which was subsequently inserted. Finally, a third bone pin was inserted using an aligned three-pin pelvic clamp, and the Mako pelvic array was securely attached.

The Mako registration was performed in enhanced mode. Reaming was performed at 53 mm, 55 mm, and 56 mm using the Mako system, followed by implantation of a 56 mm Trident2 titanium cup (Stryker Orthopaedics) without screw fixation. The femoral component of the size 6 Insignia stem (Stryker Orthopaedics) was placed, and a Delta 36 mm head was implanted after confirming dislocation stability.

**Figure 2:**
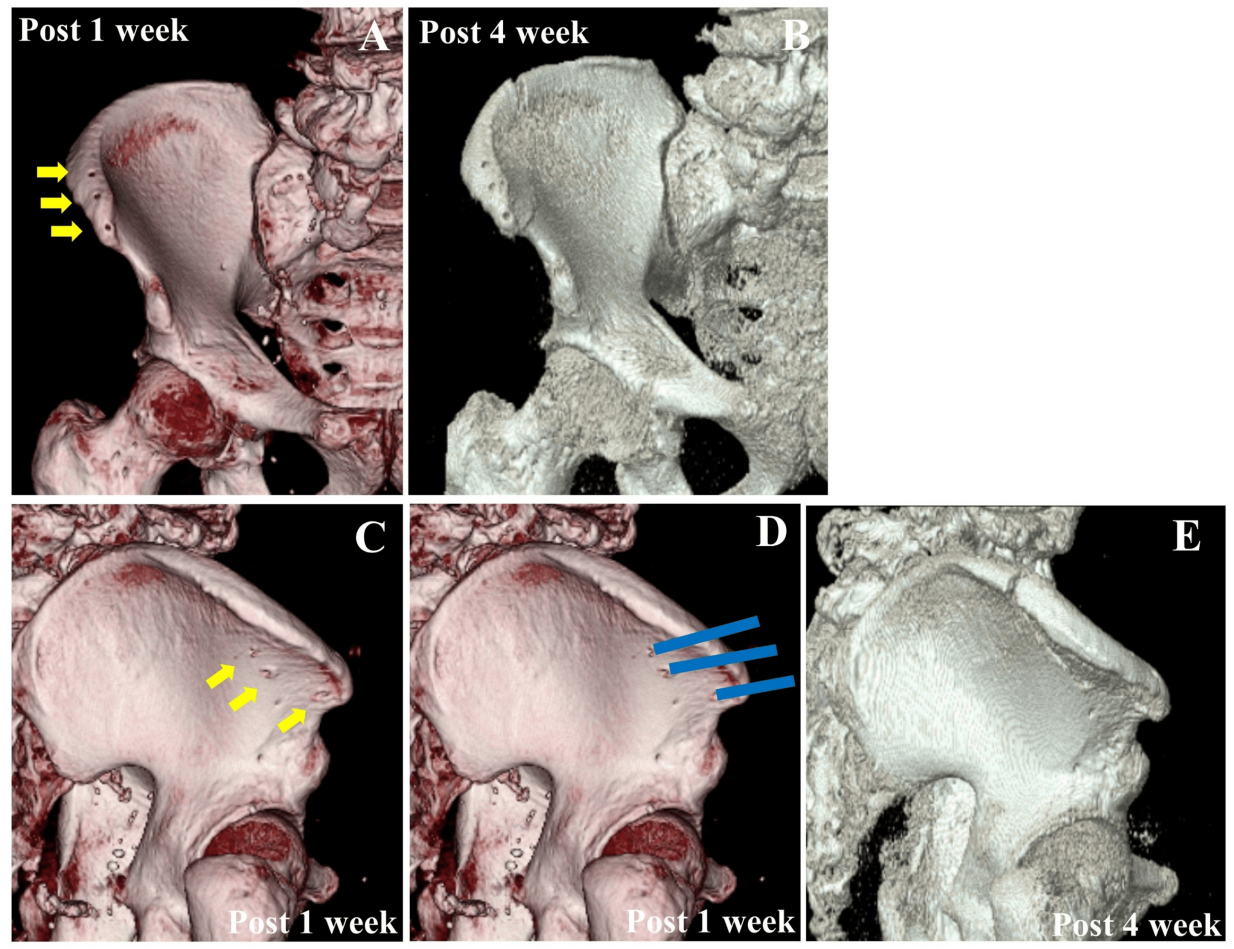
Postoperative 3D images of case one A: The 3D images of the pelvis one week postoperatively. The yellow arrows indicate the pin holes at the iliac crest. B: The 3D images of the pelvis four weeks postoperatively. C: Lateral images of the pelvis one week postoperatively. The yellow arrows indicate the pin holes at the outer cortex of the ilium. D: Lateral pelvic images one week postoperatively, with an overlay of the bone pin insertion site indicated by the blue lines; E: Lateral images of pelvis four weeks postoperatively. The fracture line extended through all three pin holes in the outer cortex of the ilium.

The bone pins were subsequently removed without evidence of loosening. The total operative time was 90 minutes, with an estimated blood loss of 500 mL. Full weight bearing was initiated postoperatively. After obtaining patient consent, a low-dose CT scan of the pelvis was performed one week postoperatively, confirming proper implant placement. The patient was discharged without pain (Figure 1B, Figures 2A-2C). Postoperative radiographic measurements showed cup alignment with a radiographic inclination of 42° and an anteversion of 22°, closely matching the intraoperative record of Mako (40° inclination, 20° anteversion). These measurements were obtained from supine pelvic radiographs using the method described by Lewinnek et al. [3].

Four weeks postoperatively, the patient presented to our hospital with right iliac pain at the site of pin insertion. The pain was characterized as moderate and dull, worsened by walking and weight-bearing activities, and relieved by rest. Radiography and CT confirmed an insufficiency fracture at the right iliac crest, where the anterior fibers of the gluteus medius were attached (Figures 1C-1D and Figures 2B-2E). The fracture line extended from the outer to the inner cortex of the iliac crest. Conservative treatment with full weight-bearing was initiated as tolerated, and the patient’s pain gradually resolved over four weeks. Bone union was confirmed at three months (Figure 1E), with no complications at six months. The Harris hip score (HHS) at postoperative three months was 95. One week postoperatively, CT images revealed bicortical fixation of all three pins and screw holes in the outer cortex of the ilium (Figure 2C). The estimated positions of the bone pin insertion, based on an analysis of the bone pinholes using reconstructed CT images obtained one week postoperatively and the 3D images at four weeks postoperatively, demonstrated the insufficiency fracture of the iliac crest through three screw holes in the outer cortex, as shown in Figure 2D and Figure 2E, respectively. The inner cortex fracture line was observed independently from these pin sites.

Case two

**Figure 3:**
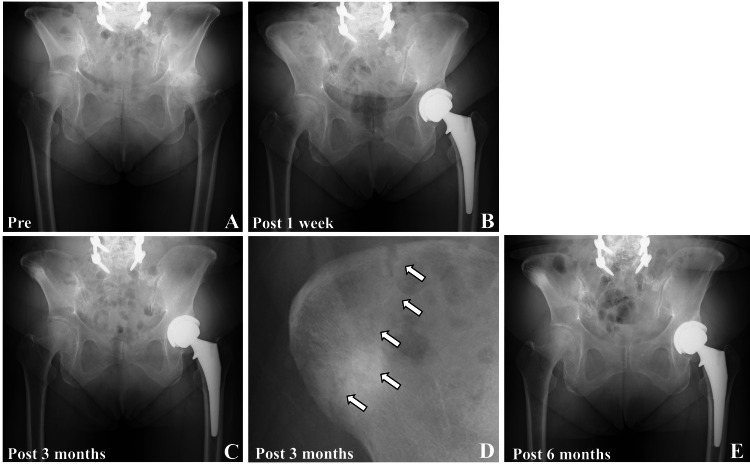
Pre and postoperative X-rays of case two A: Preoperative pelvic X-ray showing left hip osteoarthritis; B: Postoperative X-ray one week after surgery; C: X-ray at three months postoperatively showing a stress fracture of the right iliac crest; D: Enlarged view of the right ilium from panel C. The white arrow indicates the fracture line of the iliac crest. E: The X-ray taken six months postoperatively shows bone union of the iliac crest fracture without displacement.

A 79-year-old female (height 148 cm; weight 62 kg) diagnosed with left hip osteoarthritis (Kellgren-Lawrence grade 4) underwent THA (Figure 3A). She had undergone posterior interbody fusion fixation for lumbar spinal stenosis and had been receiving oral bisphosphonate therapy for approximately three years postoperatively. The preoperative BMD, assessed using the T-score, was 0.4 in the lumbar spine (L1-L4) and 0.8 in the proximal left femur. The THA was performed via the ALS approach. Three 4 mm pins were secured on the right (contralateral) iliac crest using the same procedure as in case one. Acetabular reaming with the Mako was performed at 45 mm, 47 mm, and 48 mm, followed by implantation of a 48 mm Trident2 cup without screw fixation. The femoral component of the size 4 Insignia stem was placed, and a Delta 36 mm head was implanted after confirming dislocation stability. Finally, the central pin was found to be loosened when the pins were removed. The total operative time was 105 minutes, with an estimated blood loss of 160 mL. Full weight-bearing was initiated postoperatively. After obtaining patient consent, a low-dose CT scan of the pelvis was performed one week postoperatively, and proper implant placement was confirmed with no evidence of fracture. The patient was discharged with cane-assisted walking two weeks postoperatively. Postoperative X-ray measurements at one week showed cup alignments with a radiographic inclination of 40° and anteversion of 12°, closely matching the intraoperative record of the Mako (40° inclination, 15° anteversion) (Figure 3B).

**Figure 4:**
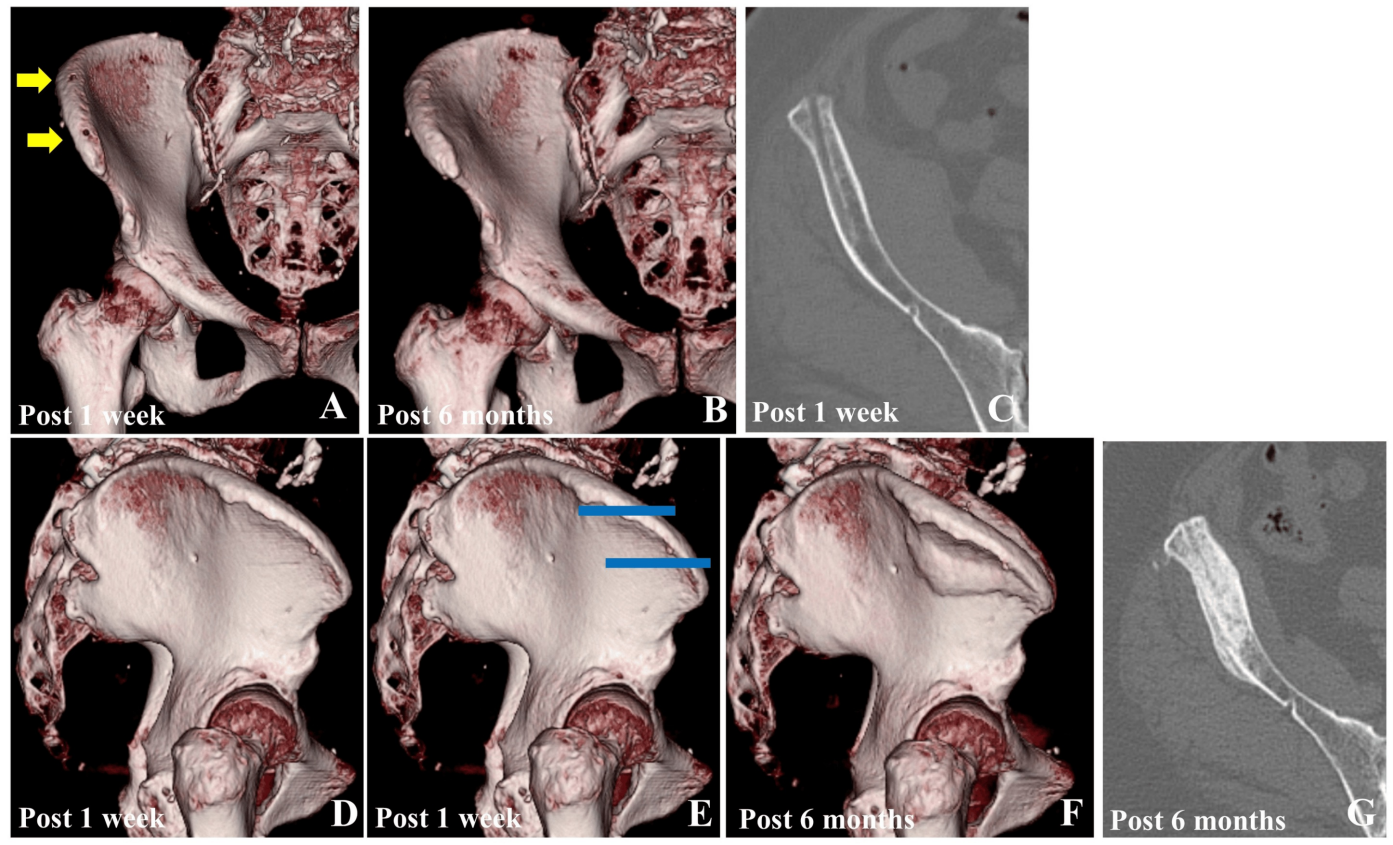
Postoperative 3D and CT images of case two A: The 3D image of the pelvis one week postoperatively. The yellow arrows indicate the insertion sites of two bone pins. B: The 3D pelvic image at six months postoperatively shows the bony union of the inner cortical layer of the ilium without displacement. C: Axial CT image of the pelvis at the level of the distal pin hole at one week postoperatively, demonstrating long transcortical fixation through the outer cortex. D: The 3D lateral pelvic image at one week postoperatively shows no visible pin holes in the outer cortex of the ilium. E: Lateral pelvic image at one week postoperatively, with an overlay of the bone pin insertion sites indicated by blue lines. F: Lateral pelvic image at six months postoperatively. The fracture line extended along the line of transcortical fixation. G: Axial CT image of the pelvis at the level of the distal pinhole six weeks postoperatively, showing the bony union of the iliac bone.

The patient experienced right pelvic pain four weeks postoperatively. Pelvic radiography was performed by the primary care physician; however, no abnormalities were detected. As the patient had no pain in the operated left hip, she did not visit our hospital. Her right pelvic pain subsided 10 weeks postoperatively. Pelvic radiography performed three months postoperatively confirmed an insufficiency fracture at the iliac crest of the pin insertion site (Figures 3C, 3D). As her pain had already improved, we carefully monitored her for six months postoperatively. Complete bone union was achieved six months postoperatively and confirmed on pelvic radiographs and CT images (Figure 3E and Figure 4B, 4F-4G). The HHS scores at three and six months postoperatively were 79 and 92, respectively. The CT images obtained one week postoperatively showed that only two of the three pinholes were visible on the iliac crest, suggesting that the central pin had been inserted into the soft tissue (Figure 4A and Figure 4D). The proximal pin was inserted in a monocortical manner. The distal pin was inserted deeply in a transcortical manner, extensively shaving the outer cortex of the ilium (Figure 4C). Figure 4D shows a lateral 3D image of the pelvis one week postoperatively without a pinhole at the outer cortex of the ilium. Figure 4E shows the estimated positions of the bone pin insertion based on an analysis of the bone pinholes using reconstructed CT images. Figure 4F shows the CT images taken six months postoperatively. The images revealed that the insertion site of the transcortical fixation of the distal pin corresponded to the fracture line of the outer cortex of the ilium. Figure 4G shows an axial CT image of the pelvis at the level of the distal pinhole six weeks postoperatively, demonstrating a bony union of the iliac bone.

## Discussion

To the best of our knowledge, insufficiency fractures at pin insertion sites have not been previously reported in robot-assisted or navigation-guided THA [2,6,8]. However, in navigation-guided total knee arthroplasty (TKA), several reports have described stress fractures in long bones such as the femur and tibia due to pinholes, with bicortical and transcortical pin fixation identified as risk factors [9,10]. Lambers et al. conducted a telephone survey of patients who underwent navigation-guided THA, reporting iliac crest pain in 30% and 2% of patients at three and 12 weeks postoperatively, respectively. However, their study did not include radiographic assessment [11]. Both patients in our study showed good recovery immediately after THA but developed contralateral iliac crest pain approximately one month postoperatively. Neither patient had a history of trauma. In terms of BMD, case one had osteoporosis, while case two had normal BMD values. However, case two involved an older female patient who had been prescribed bisphosphonate therapy following spinal surgery, and intraoperative findings suggested the presence of bone fragility. The CT images one week postoperatively showed no fractures in either case; however, contralateral iliac fractures were confirmed by pelvic X-ray at one (case one) and three (case two) months postoperatively. These findings suggest that the insufficiency fractures of the contralateral iliac crest are due to the pin fixation. A summary of the key features of both cases of insufficiency fracture of the iliac crest is presented in Table 1.

**Table 1:** Summary of key features in both cases of insufficient iliac crest fracture

Patient characteristics	Case one	Case two
Age (years) and sex	60-year-old male	79-year-old female
Body mass index (kg/m^2^)	22.1	28.3
Bone mineral density (T score)	0.7 (lumbar spine), -2.8 (proximal femur)	0.4 (lumbar spine), 0.8 (proximal femur)
Comorbidities/medical history	Hemodialysis	Bisphosphonate therapy
Insertion site of pins	Contralateral iliac crest	Contralateral iliac crest
Pin diameter/length (mm)	4/50	4/50
Type of fixation in three pins	Bicortical fixation of three pins	Monocortical, soft tissue, transcortical
Time of fracture onset	Four weeks postoperatively	Four weeks postoperatively
Time to bone union	12 weeks postoperatively	10 weeks postoperatively

In our study, postoperative CT images at one week showed that case one had all three bicortical pin fixations with the iliac bone, while case two had two monocortical pin fixations with the iliac bone, and the distal pin had a long transcortical pin fixation. Ossendorf et al. reported that bicortical pin fixation of the tibia and femur in navigation-assisted TKA could lead to postoperative stress fractures [9]. Their report was consistent with our findings. In this report, we highlight the necessity of considering insufficiency fractures even in non-long tubular bones such as the iliac crest, which are not subjected to excessive weight bearing. Jung et al. reported that transcortical pinning in navigation-assisted TKA significantly increases the risk of postoperative stress fractures. They attributed this to both a reduction in bone strength caused by bicortical drilling and a delay in bone remodeling due to thermal necrosis induced by the drilling process [8]. Consistent with their report, the insufficiency fracture observed in case two suggests that both bicortical and transcortical pin fixation can pose a risk of insufficiency fractures in the iliac crest. Based on the postoperative CT images, the bone pin insertion sites appeared to be appropriately located, suggesting that a technical error was unlikely to be the primary cause of insufficiency iliac crest fractures.

**Figure 5:**
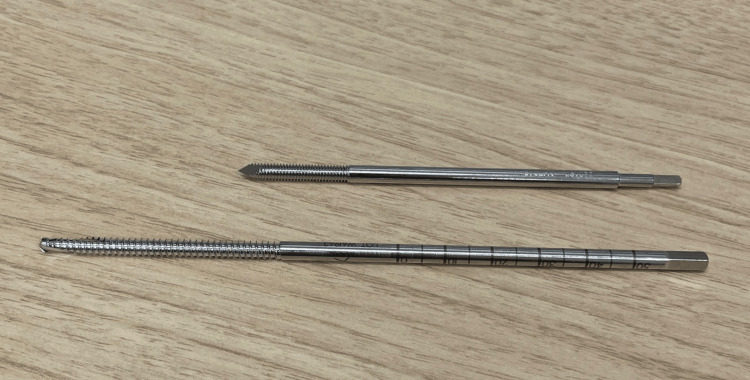
The upper pin represents the pin for CT navigation, and the lower pin represents the pin for the Mako. The pin for the Mako has a longer threaded section and a larger threaded interval than the one for CT navigation.

The orthopedic surgeon (TT) performing these procedures in this study has experience with over 100 cases of CT navigation-assisted THA and has never encountered insufficiency iliac fractures due to pin insertion. In both cases reported here, two bone pins were used for CT navigation, inserted using a freehand technique without fluoroscopic guidance. The estimated insertion depth was approximately 30 mm. Both the CT navigation and MAKO pins had the same diameter of 4 mm. Compared to CT navigation pins, MAKO pins have a longer threaded section (CT navigation vs. the Mako (25 mm vs. 50 mm)) and a larger thread interval (pitch) (Figure 5). Although there are no clear instructions regarding the depth of bone pin insertion, the longer threaded bone pins used in the Mako may increase the risk of unintended bicortical or long transcortical fixation if the surgeon attempts to fully insert the threaded section into the iliac bone. However, in patients with poor bone quality, secure pin fixation remains essential to maintain the accuracy of robot-assisted THA. To prevent insufficiency fractures while ensuring adequate fixation, we recommend inserting the pins to an appropriate depth, based on tactile feedback during drilling, regardless of the length of the threaded portion, and targeting the center of the ilium to avoid transcortical fixation. It is also important to provide patients with appropriate information regarding this potential complication. In our two cases, the bone pins were inserted into the contralateral (non-surgical) iliac crest, which may have been subjected to increased mechanical loading due to compensatory contraction of the gluteus medius and related muscles during ambulation in the early postoperative period. This factor might have contributed to the development of insufficiency fractures at the pin insertion site.

## Conclusions

In this report, we emphasize the importance of assessing bone quality and avoiding unnecessary deep insertion of the pins, regardless of the threaded length, especially when sufficient fixation is achieved based on tactile feedback during drilling. This will help prevent insufficiency iliac crest fractures. Orthopedic surgeons using the Mako system should be aware of the risk of insufficiency fractures caused by pin insertion. Increased awareness, careful surgical technique, and consideration of bone quality are essential to minimize this rare but important complication.
